# Depression in cancer patients: Pathogenesis, implications and treatment (Review)

**DOI:** 10.3892/ol.2015.2944

**Published:** 2015-02-09

**Authors:** HAMISH R. SMITH

**Affiliations:** School of Medicine and Dentistry, James Cook University, Townsville, Queensland 4811, Australia

**Keywords:** depression, psycho-oncology, psychoneuroimmunology, psychosomatics

## Abstract

Depression is a common comorbidity in cancer cases, affecting >10% of patients. A cancer diagnosis is life-changing, and is a source of considerable psychological and emotional stress. Non*-*pathological sadness may be a normal response to a cancer diagnosis, however, stress beyond the coping mechanisms of patients may result in major depressive disorder. The current review, in addition to the obvious psychosocial elements of depression, explores its biological mechanisms, including tissue damage, inflammatory mediators and the chronic stress response, and how these immune and endocrine pathways may underlie depression in cancer. Possible iatrogenic causes of depression in cancer are also explored. There is a strong need to identify and treat depression in cancer patients in order to increase quality of life and reduce mortality. The most popular clinical and potential future biochemical screening tools for depression in cancer are briefly discussed. The interventions used will vary for every patient, but may include psychosocial therapies or pharmacotherapy; however, a paucity of research on the most effective management of depression in cancer means the optimal combination of therapies is unknown. Selection of antidepressants should be carefully considered, given the common side effects of chemotherapy (such as nausea), and the necessity to avoid serious interactions, including reducing the effectiveness of chemotherapeutic drugs. The possible link between the chronic stress response, which may predispose patients to depression, and the risk of mortality from cancer is also explored. The complex interactions between the endocrine, nervous and immune systems, which continue to be elucidated, may offer the opportunity for the development of more rapid and efficacious treatments for depression in cancer in the future.

## 1. Introduction

Cancer is a life-threatening and feared diagnosis, and is a source of great distress in patients. A cancer diagnosis generates a higher sense of distress than non*-*neoplastic diseases with poorer prognoses (1). High levels of mental distress for sustained periods of time in cancer patients may lead to anxiety, depression or both (2). This mixed symptomatology is very common, with two thirds of cancer patients with depression also expressing clinically significant levels of anxiety (3).

Depression leads to a poorer quality of life (QOL) and compromises patient outcomes, with depression resulting in higher rates of mortality in cancer (4,5). A meta-analysis revealed that minor or major depression increases mortality rates by up to 39%, and that patients displaying even few depressive symptoms may be at a 25% increased risk of mortality (6). The impact of mood and mental wellbeing on cancer progression is considered important by doctors and patients, with >70% of oncologists and 85% of patients believing that mood affects the progression of cancer (7).

The rate of depression in cancer patients is thought to be up to three times higher than in the general population (2). Studies using Diagnostic for Statistical Manual of Mental Disorders (DSM) criteria (8) for major depressive disorder (MDD) have identified a variety of prevalences ranging from 2.0–43.5% (9,10), whilst palliative care wards have documented rates of depression as high as 49.0% (11). The wide range of reported prevalences may be due to differences in assessment tools, variation in the types of patients interviewed, varying age groups, varying gender proportions, inpatient status and other factors. A study by Linden *et al* (2), and a comprehensive literature review by Ng *et al* (12) detailing rates of depression in >9,000 patients, each in a variety of settings and ages, calculated the prevalence as 10.8% and 12.9%, respectively. In addition, a further 16% of patients are reported to have subclinical, yet still damaging, depression (2,5).

The site of primary cancer also influences rates of depression, with depression being most common in pancreatic and lung cancers and lowest in invasive skin cancer (2). Age also influences prevalence; evidence suggests that children and adolescents with cancer are no more depressed than healthy controls (13,14), whilst for some cancers among adults, age was inversely associated with depression (2). Gender is also a significant factor: In some cancer types female patients were found to be two to three times more likely to experience depression than males (2). Levels of psychological stress and depression also vary over the course of the disease and are highest around the time of diagnosis (2). However, rates of depression in cancer survivors five years following diagnosis were demonstrated to be comparable to the broader public, at 4% (15).

Metastases and cancer pain have also been associated with higher levels of depression (11). The prevalence of depression in patients with high levels of pain compared with low pain levels is significantly higher; one study observed that depression occurred in 33% of those in high amounts of pain, compared with 13% in those with low levels of pain, suggesting that pain may be a causative factor in depression (16).

## 2. Symptoms and diagnosis

For the diagnosis of MDD according to the DSM, the patient must have either a depressed mood or a diminished level of interest or pleasure in activities for at least two weeks. In addition, four or more of the following must be present: Significant weight change, hypersomnia or insomnia, psychomotor changes, fatigue, feelings of guilt or worthlessness, poor concentration or cognition and recurrent thoughts of death or suicide. The symptoms must also cause significant distress or impairment, and must not be a result of the physiological effects of a substance or disease (8).

The somatic presentations of depression, including fatigue, loss of appetite, weight change and poor cognition, can be dismissed by clinicians simply as symptoms of cancer or side effects of treatment, which leads to decreased detection of the disorder. In one study, appetite changes and reduced cognitive ability were found to be positively associated with anhedonia, whereas fatigue and sleep difficulties were not, even after adjusting for cancer pain and physical functioning. This suggests that reduced appetite and poor cognition may be more useful symptoms in diagnosing depression in cancer (17). Feelings of guilt and failure are also lower among depressed cancer patients, at 4%, compared with depressed but otherwise healthy patients, at 56.5% (18). Diagnosis is complex as it may be normal for some of the above symptoms to be present given the immense physical and psychological strain on patients. Patients may also battle with other behavioural changes, including sleep difficulties, poor cognition, anorexia, social withdrawal and fatigue (19,20).

### Screening

Screening is important to identify patients who are depressed and would benefit from further support (4,5). The Hospital Anxiety and Depression Scale - Depression (HADS-D) (21) is a self-administered screening questionnaire with seven questions regarding depressive symptoms, each scored by the patient from 0–3. Scores of ≥8 are indicative of probable depression, whilst scores of ≥11 are highly indicative of depression (21).

A study measuring interleukin-6 (IL-6) levels and relative diurnal cortisol variation in depressed cancer patients revealed that IL-6 levels are increased by a factor of seven (18.7 pg/ml vs. 2.7 pg/ml), whilst relative diurnal cortisol variation is decreased by a factor of six (11.7% vs. 60.6%) among cancer patients with depression compared with those without depression. Screening tests for depression in cancer patients using diurnal cortisol variation, at a cut-off value of 35%, provided the highest specificity and sensitivity, at 88% and 81%, respectively; these tests may therefore be useful in detecting depression in cancer patients (22).

## 3. Pathogenesis

Depression in cancer is a multifactorial disorder involving psychosocial, biological and even iatrogenic causes.

### Psychological and social

Depression exists on a continuum ranging from non-pathological sadness, to an adjustment disorder, to subclinical depression to major depression. Depression may result from stress that overwhelms a person’s ability to adjust to changes in life, leading to a persistently low mood, despair, anhedonia and feelings of hopelessness. Emotional stress may stem from a bleak prognosis or the extreme uncertainty that patients endure. This is compounded by the negative effects that a cancer diagnosis and treatment may have on a patient’s job, family, physical appearance, abilities, independence and finances. Those with maladaptive coping strategies, previous mental illness and poor communication with medical practitioners are at particularly high risk of developing depression (10). Strong emotional support from friends and family and an optimistic view are protective factors from developing depression (2).

### Inflammation

Evidence suggests that, in addition to the obvious emotional and psychosocial dimensions of depression in cancer, biological mechanisms may also be important. Tissue destruction by surgery, chemotherapy or radiotherapy leads to damage-associated molecular patterns (DAMPs) on damaged tissue, which bind to pattern recognition receptors (PRRs) on leukocytes, particularly macrophages, causing the expression of the transcription factor nuclear factor-κ-β (NFκβ) and the production of numerous pro-inflammatory cytokines, including interleukin-1 (IL-1), interferon-α (INF-α), IL-6 and tumour necrosis factor-α (TNF-α) (23). Ionising radiation and some chemotherapeutic agents are also able to directly stimulate NFκβ independently of tissue damage and further increase inflammatory mediator expression (24). Psychosocial stress in healthy patients has also been shown to induce NFκβ expression (25).

TNF-α, IL-1 and other cytokines have also been observed to increase the activity and expression of serotonin (5-hydroxytryptamine; 5-HT) and noradrenaline (NA) reuptake transporters, through activation of the p38 mitogen activated protein kinase (MAPK). This effectively lowers synaptic concentrations of 5-HT and NA, and can lead to depressive behaviours (26–28). Proinflammatory cytokines also increase the secretion of corticotropin releasing hormone (CRH). CRH itself can cause behavioural changes including those observed in depression (29). Furthermore, these cytokines reduce levels of neural growth factors, such as brain-derived neurotrophic factor (BDNF), which is key for neurogenesis. Low levels of BDNF and neurogenesis have been implicated in the pathogenesis of depression (30).

In non-human primate studies, INF-α has been observed to decrease the expression of dopamine (DA)-2 receptors and reduce striatal DA release, leading to feelings of anhedonia. Although the mechanisms behind this and any possible pharmacological strategies remain to be elucidated (31), INF-α also results in decreased conversion of phenylalanine to tyrosine, which causes reduced downstream formation of DA in the brain and, potentially, depressive symptoms (32).

Pro-inflammatory cytokines, particularly TNF-α, have been shown to increase the activity of an enzyme called indolamine-2,3-dioxygenase (IDO), which degrades tryptophan. Tryptophan is the chemical precursor to 5-HT; therefore its destruction results in reduced 5-HT levels (33). It is well established that tryptophan levels are decreased relative to monoamine levels in the brains of depressed patients (34). Induction of IDO also causes the formation of neurodegenerative tryptophan catabolites (TRYCATs). These TRYCATs, including kynurenine and quinolinic acid, may also be important in depression in cancer, as they are proinflammatory and neurotoxic in the brain. Quinolinic acid is a potent N-methyl-D-aspartate (NMDA) receptor agonist and is able to induce lipid peroxidation in neurons by excitotoxicity, whilst kynurenine has been demonstrated to be anxiogenic (35). In severe depression, levels of quinolinic acid have been observed to be raised in several regions throughout the anterior cingulate cortex. Neurotoxicity and degeneration, particularly of the hippocampus, have been implicated in the development of depression (36).

### Chronic stress

Living with the physical and psychological burden of a cancer diagnosis and treatment is a form of chronic stress. During stress, the sympathetic nervous system (SNS) is stimulated, the parasympathetic nervous system (PSNS) is inhibited and the hypothalamic-pituitary-adrenal (HPA) axis is activated (26,27,37–39). The stimulation of the HPA-axis should lead to the downstream release of endogenous glucocorticoids, potent anti-inflammatories which normally inhibit pro-inflammatory cytokine production. However, PRR activation by DAMPs causes the intracellular glucocorticoid receptor (GCR) in leukocytes to be inactivated by p38 MAPK; the anti-inflammatory effect of glucocorticoids is therefore attenuated, and cytokine expression is increased (23,40). Proinflammatory cytokines may also lead to decreased sensitivity in the remaining activated GCRs, resulting in a further decrease in anti-inflammatory activity of glucocorticoids and increased cytokine production (41). Additionally, the increased NA, released due to chronic SNS stimulation, binds to adrenergic receptors on macrophages, which causes further NFκβ activation and cytokine expression, leading to further reductions in 5-HT and NA concentrations (27,28). Usually PSNS activity should inhibit the production of pro-inflammatory cytokines; acetylcholine binds to nicotinic receptors on leukocytes and inhibits the production of NFκβ (38). However, the PSNS is inhibited in a chronic stress state, instead leading to increased cytokine formation, which acts to further alter neurotransmitter levels (38,39).

### Medications

Some medications have been implicated in causing depression-like symptoms in cancer patients. The DA receptor-2 antagonist haloperidol, occasionally used in the treatment of chemotherapy-associated nausea, reduces dopaminergic transmission in the brain and has been linked to the development of depressive symptoms (42). Immunotherapy agents, including INF-α, used in some cancers have been reported to cause depression in up to 50% of patients by the mechanisms explained above (26–28).

## 4. Implications

### Chemotherapy and the health care system

In a cohort study investigating breast cancer outcomes in patients with depression, only 51% of the study group (breast cancer patients with depression) accepted and commenced chemotherapy, compared with 92% of the control group (breast cancer patients without depression) (4). Depression affects treatment participation, causes poorer outcomes and results in higher mortality (4,5).

Depression causes a greatly diminished QOL in cancer patients by worsening physical symptoms, and increases the negative impact on patients and their families throughout the course of the disease (2,43). For example, during treatment for breast cancer, patients with higher pre-chemotherapy levels of fatigue, depression and sleep disturbances suffered increased levels of these symptoms during treatment, which had severe and adverse effects on their QOL (44). Depression in cancer patients increases the length of hospital stay and resource use, increasing health expenditure (45). Depressed cancer patients are also at a higher risk of suicide compared with the general public (46).

### Cancer progression and depression

Depressed cancer patients are at increased risk of mortality compared with non-depressed patients (5). Although treatment non-participation accounts for some of the increased risk, evidence from animal studies and human studies suggests that the chronic stress response, which may contribute to the development of depression in cancer, may also contribute to increased cancer invasiveness, reduced tumour surveillance by the body, increased angiogenesis, reduced tumour suppressor gene activity and reduced cellular apoptosis (6,47–51).

### Immune modulation

Numerous immune mechanisms are thought to underlie the increased risk of mortality of depressed patients compared with non-depressed patients (6). Depression in otherwise healthy adults reduces the number of circulating natural killer (NK) cells, which normally have a tumour surveillance role, and the same is predicted to apply in cancer patients (47). In ovarian cancer patients, depressed and anxious moods were associated with significantly impaired T helper 1 cell levels, cytotoxic T lymphocyte levels and interferon-γ (INF-γ) secretion in both the microenvironment of the tumour and peripheral blood (48). In one murine study, which involved exposing stressed and unstressed groups of ultraviolet radiation-sensitive mice (SKH1 hairless mice), the chronically stressed group were found to be at higher risk of developing squamous cell carcinoma than the non-stressed group. The stressed group had a shorter latency prior to the development of the first tumour, lower INF-γ expression, more infiltrating and circulating levels of regulatory T cells and reduced T helper cell infiltration (49). Therefore, chronic stress also impairs the immune system’s ability to fight cancer by reducing anti-tumour lymphocyte and NK cell activity and increasing immunosuppression.

### Gene modulation

In another murine study, chronic stimulation of the SNS during chronic stress was observed to interfere with the p53 tumour suppressor gene. The HPA axis activation was also thought to lead to elevated glucocorticoids with systemic increases in the expression of p53 inhibitors (namely MDM2). The effects of chronic stress on p53 reduces its normal critical tumour suppressor and anti-angiogenic roles (50). Chronic stress in murine prostate cancer models have also implicated catecholamines in promoting tumour growth by phosphorylating and inactivating the proapoptotic molecule Bcl-2-associated death promoter (51).

### Angiogenesis and invasiveness

In an ovarian cancer model, chronic stress led to higher levels of tissue catecholamines, the downstream increased expression of angiogenic factors, (including vascular endothelial growth factor), and increased formation of pro-invasive enzymes (such as matrix metalloproteinase-2 and -9) by protein kinase A stimulation (51). Chronic stress may stimulate angiogenesis and increase invasiveness of some tumours, leading to increased tumour burden.

## 5. Management

### Rationale

There is a requirement to effectively treat depression in cancer patients in order to improve QOL and survival (4,5). It has been demonstrated that the treatment and improvement of depression in metastatic breast cancer, and improvement of depressive symptoms within the first year, significantly prolongs median survival time by 28.5 months compared with patients that experienced an increase in depressive symptoms (52). Both psychosocial interventions and pharmacotherapy are effective in treating depression in cancer, but the optimal combination and delivery of treatments are unknown and further research is required (53). The management of depression is likely to be different in each patient.

### Medications

The two main classes of medication for depression in cancer are tricyclic antidepressants (TCAs) and selective serotonin reuptake inhibitors (SSRIs) (12). The exact mechanisms of action for these drugs has not been fully established, however, they are thought to cause adaptive changes in the brain and, over time, increase 5-HT neurotransmission leading to an improved mood (26,54).

TCAs have more adverse effects and a higher risk of overdose compared with SSRIs. However, a meta-analysis found no recommendation for one antidepressant type over another in cancer due to a lack of research (55). The prescription of SSRIs must be carefully considered in patients receiving chemotherapy or radiotherapy, as SSRIs can often worsen emesis and nausea (53), whilst the anticholinergic effects of TCAs may worsen delirium associated with chemotherapy (26,53).

### Terminal patients

The use of conventional antidepressants in patients with terminal cancer may be unwise due to the delayed mode of action of these drugs (12,53,54). An observational study supported this notion, concluding that currently prescribed antidepressants have little effect on improving depression in terminally ill depressed cancer patients, as measured by depression scores (56). In one trial, methylphenidate provided moderate to marked improvement in depression symptoms in 73% of depressed oncology patients within two days; this drug may therefore be an effective alternative to conventional antidepressants (57). However, whilst trials of methylphendidate have been promising, physiological tolerance quickly develops and doses must be increased (57,58). Ketamine has recently been studied for its rapid and effective antidepressant effects, due to its antagonism of NMDA receptors (58). Ketamine has been suggested as a treatment of depression in terminally ill cancer patients where rapid reversal of depression is vital. A trial in a single patient with advanced cancer, however, exerted initially positive but unsustainable effects; larger randomised trials are necessary to assess the role of ketamine in treating depression in terminal patients (59).

### Interactions

Chemotherapeutics may interact with antidepressants and cause nervous system toxicity, by reducing the metabolism of the antidepressants or by additive effects of the cancer drugs themselves. Some antidepressants may reduce the efficacy of chemotherapeutic drugs. A common example of this is the interaction of certain SSRIs (including fluoxetine and paroxetine, which are concurrently prescribed in 20–30% of breast cancer patients) with tamoxifen, which reduces the metabolism of tamoxifen to its active metabolite, endoxifen, by inhibiting the hepatic CYP2D6 enzyme. This reduces the effectiveness of the drug and increases the risk of breast cancer relapse (60,61). Other SSRIs and herbal preparations containing hypercium (St John’s Wort) may induce the CYP3A4 enzyme, which may lead to a reduction in levels of hepatically excreted anticancer agents, or an increase in the levels of drugs activated by CYP3A4 (62). Cancer drugs commonly have a narrow therapeutic range, therefore it is essential to avoid altering their levels. Great care must be taken in avoiding drug interactions (63).

### Non-pharmacological

Positive patient-physician relationships and communication markedly reduces levels of distress in patients (15). In addition patients may benefit from a range of approaches, including relaxation strategies, psychoeducation, cognitive behavioural therapy (CBT), problem solving therapy (PST) and acceptance and commitment therapy (ACT) (64–68).

Relaxation strategies, including meditation and progressive muscle relaxation, enable patients to relieve mental and physical tension, thereby reducing stress, and have been observed to improve depression and QOL in cancer patients (64,68). Psychoeducation may be used to build knowledge and coping strategies with regard to cancer, reducing uncertainty and anxiety. CBT identifies and equips patients with the skills to overcome maladaptive thought patterns and encourages emotional readjustment. Both psychoeducation and CBT have been demonstrated to be effective in improving depressive symptoms and QOL (65,68). PST focuses on creating, implementing and appraising solutions for the manageable problems affecting patients, including relationships and financial problems. PST may improve psychological outcomes and QOL in depressed patients (66). ACT teaches patients how to tolerate difficult thoughts without being overwhelmed or dominated by them and develops psychological flexibility. ACT has been demonstrated to be equivalent to CBT in terms of its effect on mood and QOL (68).

Exercise therapy may also be effective in providing modest relief from depression in cancer patients. A meta-analysis concluded that, although there was a scarcity of research using depression as the primary endpoint, exercise may lower pain and improve fatigue and QOL among cancer survivors (69).

## 6. Conclusion

Depression remains an under-recognised comorbidity in cancer patients, with major implications on patient suffering, mortality and healthcare expenditure. Depression in cancer is markedly different from depression in healthy individuals, and involves a unique symptomatology and a strong biological aetiology. In the future, novel anti-inflammatory drugs may be able to provide more rapid and effective relief compared with current antidepressant therapy, by targeting the extensive inflammatory and endocrine pathways thought to underlie depression in cancer patients. At present, however, further research is required to identify the most effective combinations of pharmacological and psychosocial treatments for depression in cancer.
